# The Eat-Out-to-Help-Out incentive: A trigger for gastrointestinal infections in England, 2020?

**DOI:** 10.1017/S0950268825100848

**Published:** 2025-12-26

**Authors:** Reece Jarratt, Helen Clough, Ewan Wilkinson, Roberto Vivancos, Valérie Decraene

**Affiliations:** 1 United Kingdom Health Security Agency, London, UK; 2 Institute of Infection, Veterinary and Ecological Sciences, University of Liverpool, Liverpool, UK; 3 National Institute for Health and Care Research Health Protection Research Unit in Gatrointestinal Infections, Norwich, UK; 4 Institute of Medicine, University of Chester, Chester, UK; 5 National Institute for Health and Care Research Health Protection Research Unit in Emerging and Zoonotic Infections, Liverpool, UK; 6 Warwick Medical School, University of Warwick, Coventry, UK

**Keywords:** Food-borne infections, *Campylobacter*, *Escherichia coli* (*E. coli*), *Salmonella*, Change point

## Abstract

Our study assessed the link between gastrointestinal (GI) infections in England and the Eat Out to Help Out scheme (EOHO), a government subsidy created to encourage people to eat out during COVID-19 pandemic (03–30 August 2020). We studied national laboratory data between January 2015 and December 2020. We used time series change point analysis to see if there were shifts in reported cases of specific GI infections (*Campylobacter* spp., *Escherichia coli* O157, and non-typhoidal *Salmonella* spp.) associated with the timing of the scheme. Our analysis uniquely applied the Pruned Exact Linear Time method, with generalized linear models to a national dataset of GI infections. This revealed increases in cases closely aligned to the timing of the easing of COVID-19 restrictions, prior to the introduction of the EOHO scheme. Our study showed the scheme had no measurable impact, as there was no significant change on reported cases. Substantial reductions in cases after the first lockdown, followed by an increase as restrictions were phased out, show the wider impact of COVID-19 control measures, for example, public information campaigns aimed at improving hand-hygiene. These findings highlight the complicated interactions between COVID-19 control measures, the public’s behaviour, and the spread of GI infections.

## Introduction

Gastrointestinal (GI) infections result in health and public health challenges globally, causing considerable morbidity and placing a burden on healthcare systems [1, 2].

In England, an estimated 17 million GI cases occur annually [3], resulting in a societal cost of more than £9 billion, primarily due to loss of earnings [4]. These infections follow seasonal patterns, with bacterial pathogens peaking in summer months, linked to increasing temperatures and overseas travel [5].

Foodborne infections account for an estimated 2.4 million GI cases annually in England [4]. Up to 80% of reported *Campylobacter* spp., *Salmonella* spp., and Shiga-toxin producing *Escherichia coli* (including O157) infections can be attributed to contaminated food products [6, 7].

Deprivation influences exposure to risks of foodborne infections, with lower rates of *Campylobacter* and non-typhoidal *Salmonella* reported in more deprived areas [8]. This trend may partly reflect reduced opportunities for international travel and dining out, the former being a key risk factor for the development of GI infections and more common in affluent populations [9]. However, underreporting due to disparities in healthcare-seeking behaviour within deprived populations may distort the true incidence [10].

Geographic location influences infection patterns. Urban areas present higher exposure risks due to the dense populations and non-compliant food establishments [11]. Rural areas report more *Campylobacter* cases, likely from increased environmental exposure to livestock excretions [12]. However, this may reflect deprivation classification rather than true geographic transmission differences [13].

Due to the emergence of COVID-19, governments worldwide introduced non-pharmaceutical interventions (NPIs) to reduce the transmission and burden of the virus. These interventions ranged from social distancing, closure of businesses, international-travel restrictions, and an enforceable nationwide ‘lockdowns’ [14, 15].

Such interventions, coupled with improved hand hygiene through public health information campaigns, significantly reduced rates of endemic communicable diseases, as transmission routes were removed or interrupted [9]. For GI infections this was clearly demonstrated, most notably in infections driven by person-to-person transmission. The reductions in social interactions halted the spread of infections [16].

A national lockdown left the hospitality sector financially vulnerable. Over 80% of businesses were temporarily closed [17], resulting in consumer spending in the hospitality sector falling by 56% in spring 2020 compared to pre-pandemic levels [18]. In response, the UK government introduced a fiscal policy which aimed to stimulate demand for the sector through the introduction of the eat out to help out scheme (EOHO). The scheme directly subsidized 50% of cost of meals and non-alcoholic drinks in participating venues on Mondays to Wednesdays between 3 August 2020 and 31 August 2020 [19]. The EOHO scheme was the first initiative globally to encourage individuals to dine out following the gradual easing of national lockdown regulations (10 May 2020), which allowed restaurants to reopen on 04 July 2020 [15].

The pandemic brought significant workforce challenges for the hospitality sector, with 18% of furloughed jobs originating from the industry, resulting in difficulties in retaining and recruiting trained staff [20, 21]. Together with understaffing, high customer volumes have been cited by food workers as a factor to maintaining food safety practices [22]. During the EOHO scheme, there was marked increase in restaurant visits, exceeding volumes during the same period in the previous year [23]. Meanwhile, routine hygiene inspections in England decreased by approximately a third during the pandemic compared to pre-pandemic levels [21]. Together, these factors provided opportunities for lapses in compliance to safe food handling and preparation practices, as well as increased exposure to foodborne pathogens.

Surveillance of GI infections during events that may have increased public dining behaviours (e.g., 2012 London Olympics and 2022 Birmingham Commonwealth Games) found no outbreaks or public health incidents explicitly linked to dining activities [24, 25]. Unlike sporting or cultural events, EOHO was unique in its scale, intensity, and duration. By its design, it aimed to create a significant shift in dining behaviours, differing from other temporary influences.

To date, there has been no analysis of the effects of the EOHO scheme other than assessing its impact on the incidence of COVID-19 infections [23]. Our study aimed to assess if the EOHO scheme was associated with changes in the incidence of selected GI infections during the COVID-19 pandemic in 2020 among residents of England.

## Methods

We conducted a retrospective ecological study by performing secondary analyses on routinely collected national data.

Foodborne pathogens, including *Campylobacter* spp., non-typhoidal *Salmonella* spp., and *E. coli* O157, are lawfully notifiable [26]. Laboratories in England with a primary diagnostic role are required to report cases of these pathogens to the UK Health Security Agency (UKHSA).

Our study population consisted of all residents in England who had a laboratory diagnosis of one of the three aforementioned GI infections, recorded between 1 January 2015 and 31 December 2020. Laboratory diagnoses were reviewed to ensure accurate case number calculations. Multiple diagnoses for the same individual, were grouped as a single infection episode within a pathogen-specific episode length (28 days for *Campylobacter* spp., 43 days for *E. coli* O157, and 93 days for non-typhoidal *Salmonella* spp.). Individuals were grouped using a combination of NHS number, date of birth, name, and sex. Diagnoses reported outside the specified episode length were classified as reinfections, representing new cases.

Data were collected from UKHSAs routine laboratory surveillance system (Second-Generation Surveillance System). English Index of Multiple Deprivation (2019; IMD) [27] and urban–rural residency classification (2011) [28] was linked to laboratory notifications on lower layer super output areas [29].

### Statistical analysis

A weekly mean of cases was calculated for each GI infection for each of the five years (2015–2019) and presented with 95% confidence intervals. The weekly mean provided a comparator for cases registered in 2020 and was examined to provide insights into seasonality of each pathogen.

Time-series data (2015–2019) were decomposed by infection into trend, seasonal, and residual components. An additive decomposition was applied to visually inspect seasonal patterns. The long-term trend was estimated using seasonal-trend decomposition based on Loess (STL) [30] to obtain a more robust estimate.

A generalized linear model (GLM) was produced for each infection using negative-binomial regression to predict the number of cases. Each infection was further assessed for evidence of a trend using time as a predictor within the model. Seasonality was addressed via the addition of sine and cosine pairs and construction of periodograms. The autocorrelation and partial-autocorrelation functions were plotted to identify if the number of laboratory notifications were correlated with previous values in time series. Lag terms were added to the model to address autocorrelation. Likelihood ratio tests [31] were used to determine if additional predictors provided a significantly better fit to the observed data. A stepwise selection procedure based on the Akaike Information Criterion [32] was applied to determine an appropriate parsimonious model for each infection.

Additional GLM models were developed using Poisson fit and a log-link function to predict the rate of cases of each infection, stratified by urban–rural residency and IMD. These models utilized the log-transformed population of each stratum as offset variables.

Sudden deviations between the observed cases and each of the model’s predictions were examined to identify any unexplained factors influencing the incidence of laboratory notifications (change points). We used the pruned exact linear time method [33], assessing both the mean and variance of the deviations, to identify change points.

Specifically, we assessed whether these change points corresponded to the introduction of the national lockdown or the EOHO scheme. If a change point was identified at these time points, an interruption was added to the model. If no change points were identified corresponding to the EOHO scheme, we added a step change for the period following the beginning of the scheme (3 August 2020) to assess the schemes impact on predicting cases.

Statistical analyses were performed using R (version 4.3.2) [34]. A value of *p* < 0.05 was taken as statistically significant.

## Results

There were 309,466 *Campylobacter* spp. infections, 42,018 non-typhoidal *Salmonella* spp. infections and 3,282 *E. coli* O157 infections, notified across England between 1 January 2015 and 31 December 2020, combining the pre-pandemic and pandemic phases. The majority of cases were notified pre-pandemic (1 January 2015 and 31 December 2019), accounting between 86% and 90% of cases across all three infections (Table 1). Cases from residents of urban areas formed between 69% and 84% of notifications for all three infections. The highest proportion cases for both *Campylobacter* spp. (23%) and *E. coli* O157 (26%) were from individuals living in the most deprived IMD quintile. In contrast, the highest proportion of cases for non-typhoidal *Salmonella* spp. (22%) were from those residing in the least deprived IMD quintile notified during 2020 (Table 1).

**Table 1.** Demographics of the population with gastrointestinal infections captured in England between 1 January 2015 and 31 December 2020.

Period	*Campylobacter* spp. *n* (%)		*Escherichia coli* O157 *n* (%)		non-typhoidal *Salmonella* spp. *n* (%)	
	2015–2019	2020	2015–2019	2020	2015–2019	2020
Total	266,521 (86)	42,945	2,974 (90)	308	37,989 (90)	4,029
Residency						
Urban	208,089 (78)	33,414 (78)	2,318 (78)	212 (69)	32,065 (84)	3,330 (83)
Rural	58,432	9,531	656	96	5,924	699
IMD						
1	40,785 (15)	7,049 (16)	404 (14)	35 (11)	7,635 (20)	877 (22)
2	48,236 (18)	8,034 (19)	577 (19)	52 (17)	7,925 (21)	850 (21)
3	56,437 (21)	9,174 (21)	611 (21)	66 (21)	7,837 (21)	866 (21)
4	60,059 (23)	9,338 (22)	653 (22)	76 (25)	7,362 (19)	782 (19)
5 (most deprived)	61,004 (23)	9,350 (22)	729 (25)	79 (26)	7,230 (19)	654 (16)

**Figure 1.**
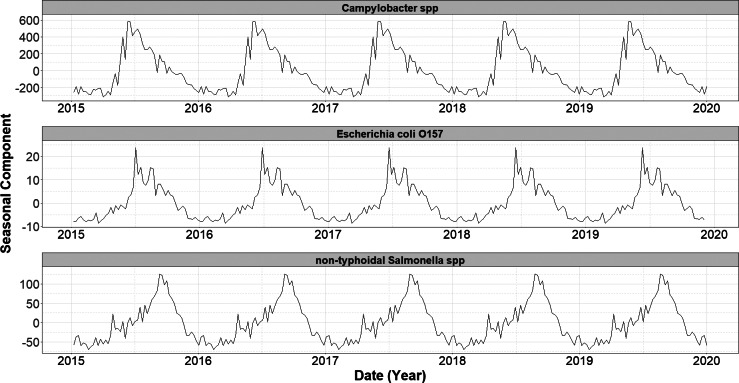
Seasonal component of decomposed additive time series of weekly laboratory confirmed gastrointestinal infections in England (2015–2019), by infection.

**Figure 2.**
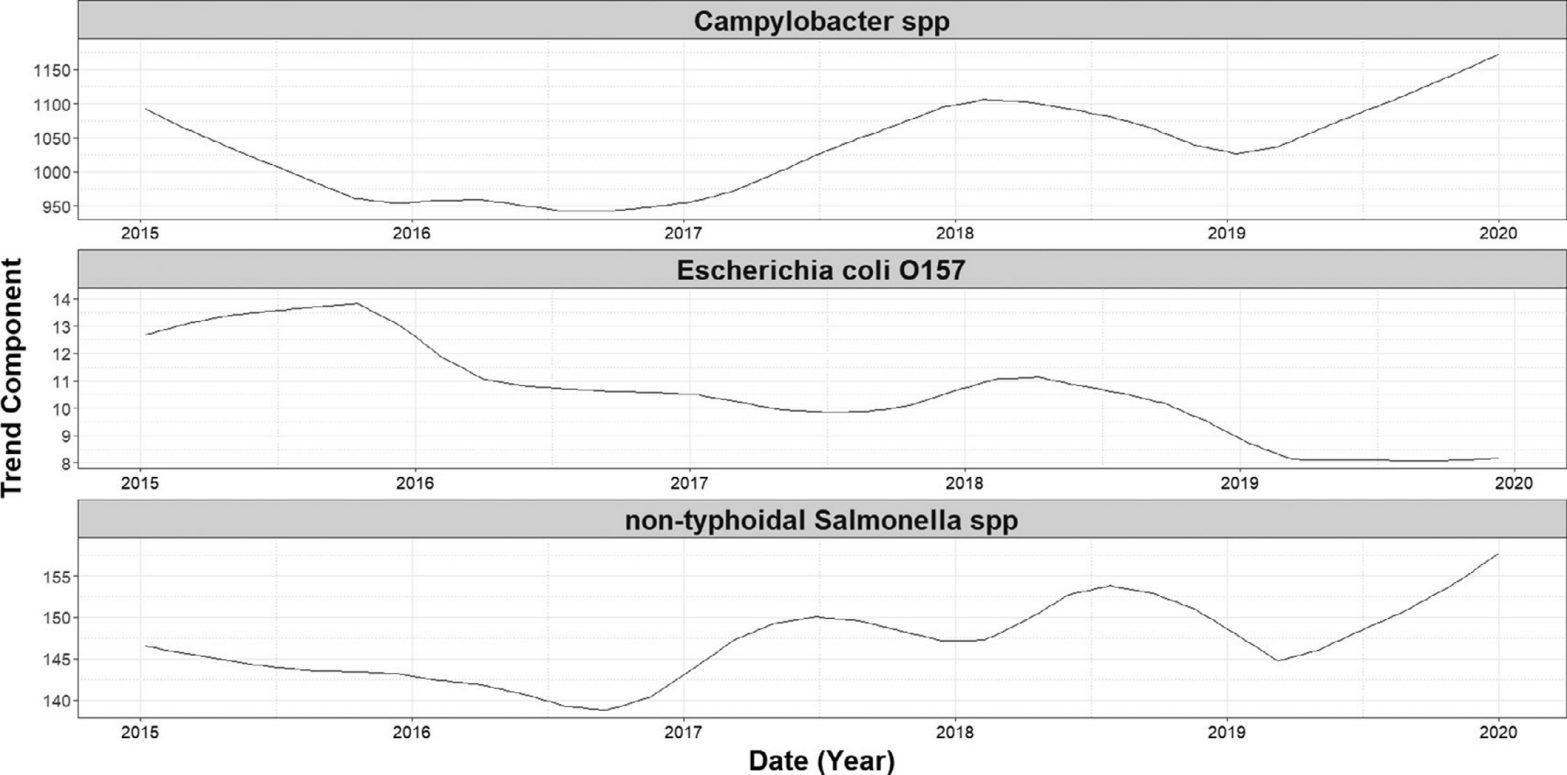
Long-term trend components of weekly laboratory confirmed gastrointestinal infections in England (2015–2019), by infection. Trend components were derived using Seasonal-Trend Decomposition based on LOESS (locally estimated scatterplot smoothing; SLT), with a periodic seasonal window to account for annual patterns.

Over the study period (1 January 2015 and 31 December 2020), we detected a significant decreasing trend in the number of cases of *E. coli* O157 (−0.002, 95% CI: −0.003 to −0.002, *p* = <0.001) (Figure 1). No significant trends were observed in case numbers of both *Campylobacter* spp. and non-typhoidal *Salmonella* spp. over the same period. A single seasonal peak was observed during the summer months for all three infections. The seasonal peak for non-typhoidal *Salmonella* spp. occurred 12 weeks later (Figure 2) than for the other two infections.

**Figure 3.**
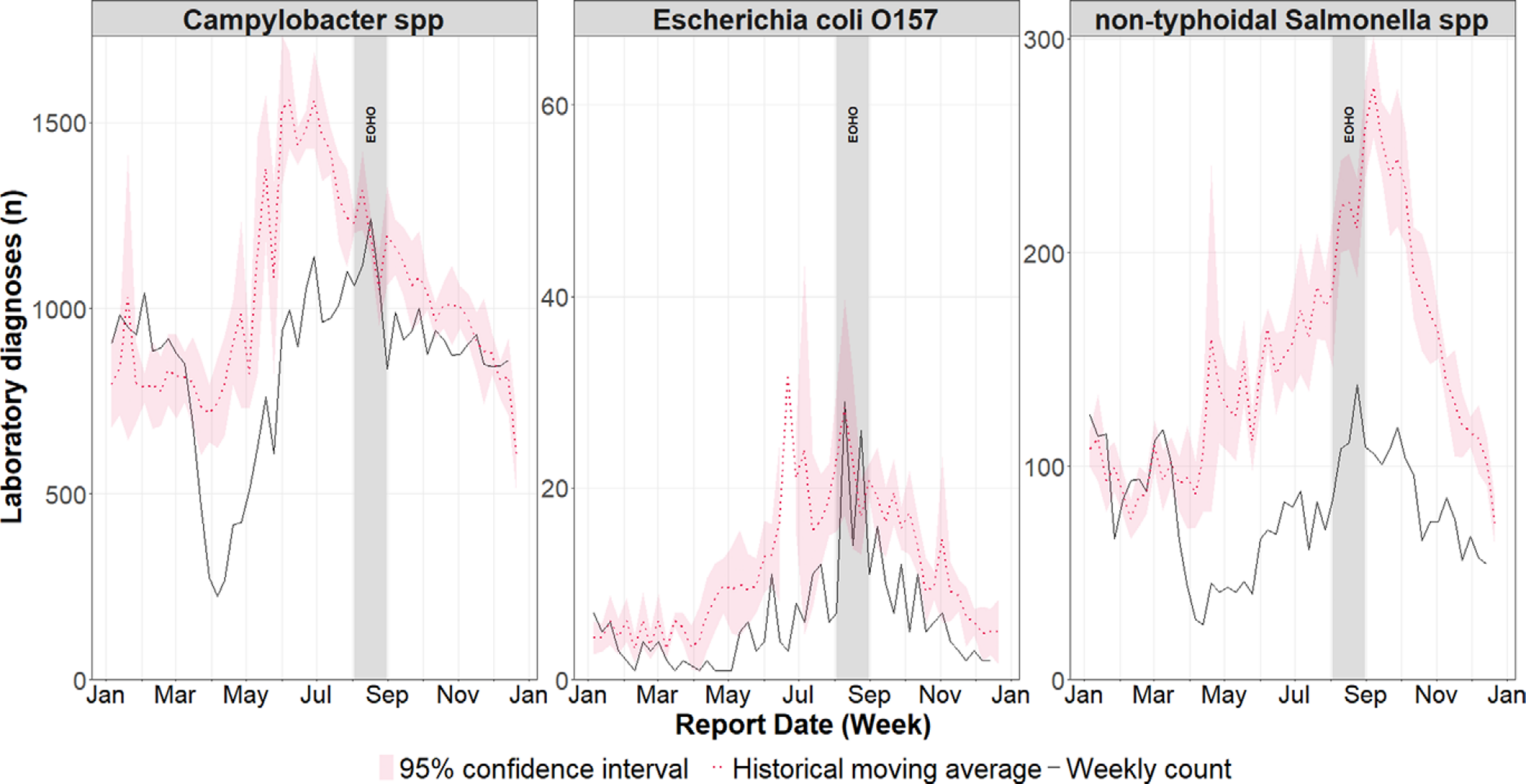
Weekly incidence of laboratory confirmed gastrointestinal infections in England during 2020, compared to the historical weekly average (2015–2019) with 95% confidence intervals.

Cases across all infections in 2020 were lower than the historical mean over the previous five years for the majority of weeks in 2020 (between 69% and 79%). The lowest number of cases was observed between weeks 7 and 20 (10 February 2020 and 17 May 2020) (Figure 3). Cases of *Campylobacter* spp. and *E. coli* O157 did increase to levels consistent with the historical mean during summer (between 10 August 2020 and 30 August 2020), corresponding to the period of EOHO. In contrast, over the same period, non-typhoidal *Salmonella* spp. cases remained substantially below the historic mean.

We did not find any significant association between the implementation of the EOHO scheme and a change in the number of cases for any of the infections (Table 2).

**Table 2.** Coefficient estimates from a negative binomial regression model examining the impact of the ‘Eat Out to Help Out’ scheme on selected gastrointestinal infections in England in 2020

Infection	Estimate	95% confidence interval		*P*-value
		Lower	Higher	
*Campylobacter* spp.	−0.028	−0.092	0.038	>0.05
*Escherichia coli* O157	0.025	−0.195	0.242	>0.05
Non-typhoidal *Salmonella* spp.	−0.187	−0.313	−0.061	<0.01

**Figure 4.**
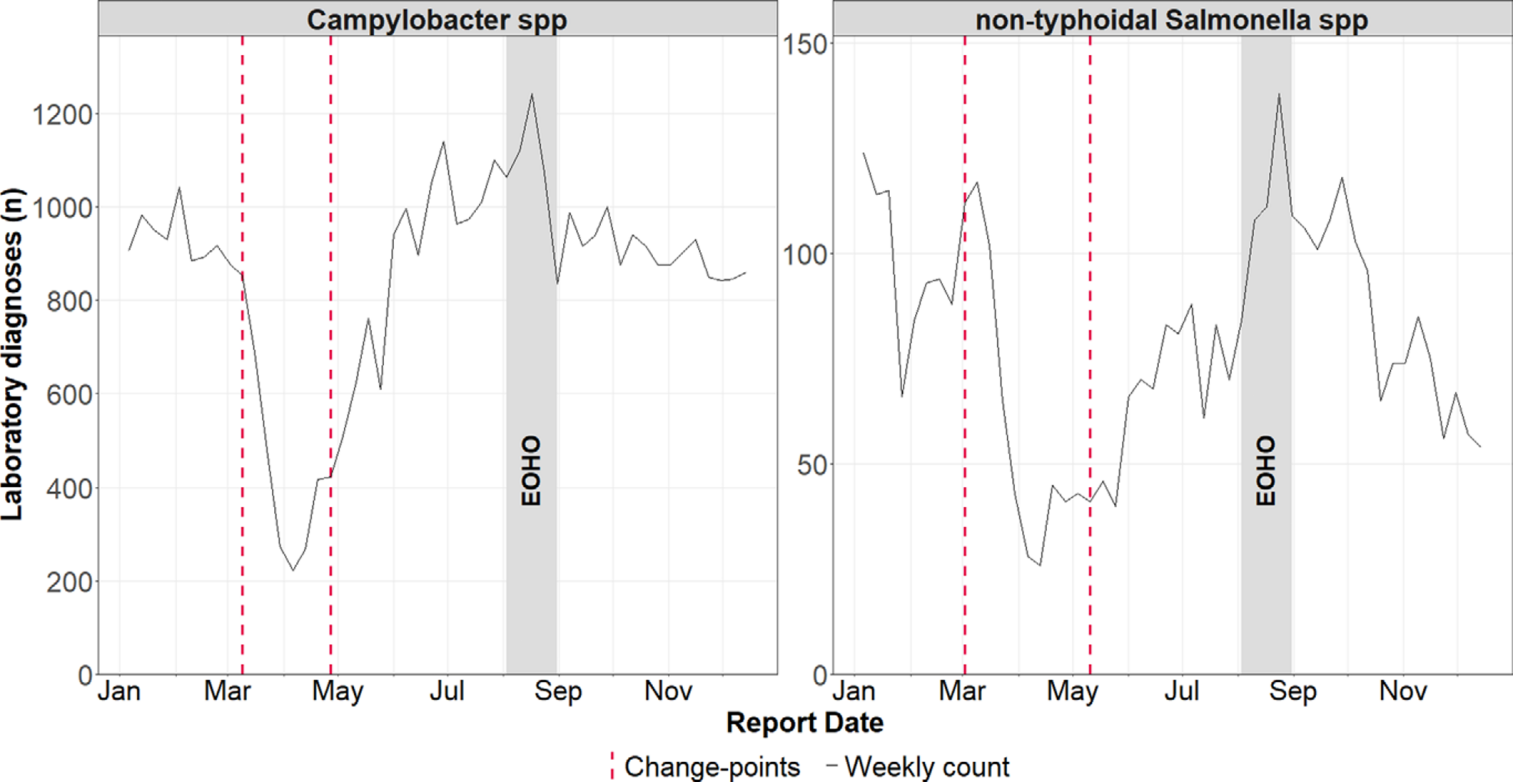
Weekly laboratory confirmed *Campylobacter* spp. and non-typhoidal *Salmonella* spp. infections in England during 2020, with identified change-points (detected using the Pruned Exact Linear Time method). Change-points reflect shifts in the mean and variance of residual deviations between observed counts and expected counts from a negative binomial generalized linear model.

Our change point analysis showed distinct shifts in the incidence of *Campylobacter* spp. and non-typhoidal *Salmonella* spp. in 2020. A significant reduction in cases occurred between week 10 (02 March 2020) and week 11 (09 March 2020), followed by a subsequent increase between week 18 (27 April 2020) and week 20 (17 May 2020) (Figure 4). No significant change points were detected for *E. coli* O157.

The analysis confirmed there were no unexpected changes in the incidence of any of the infections during the EOHO scheme.

**Figure 5.**
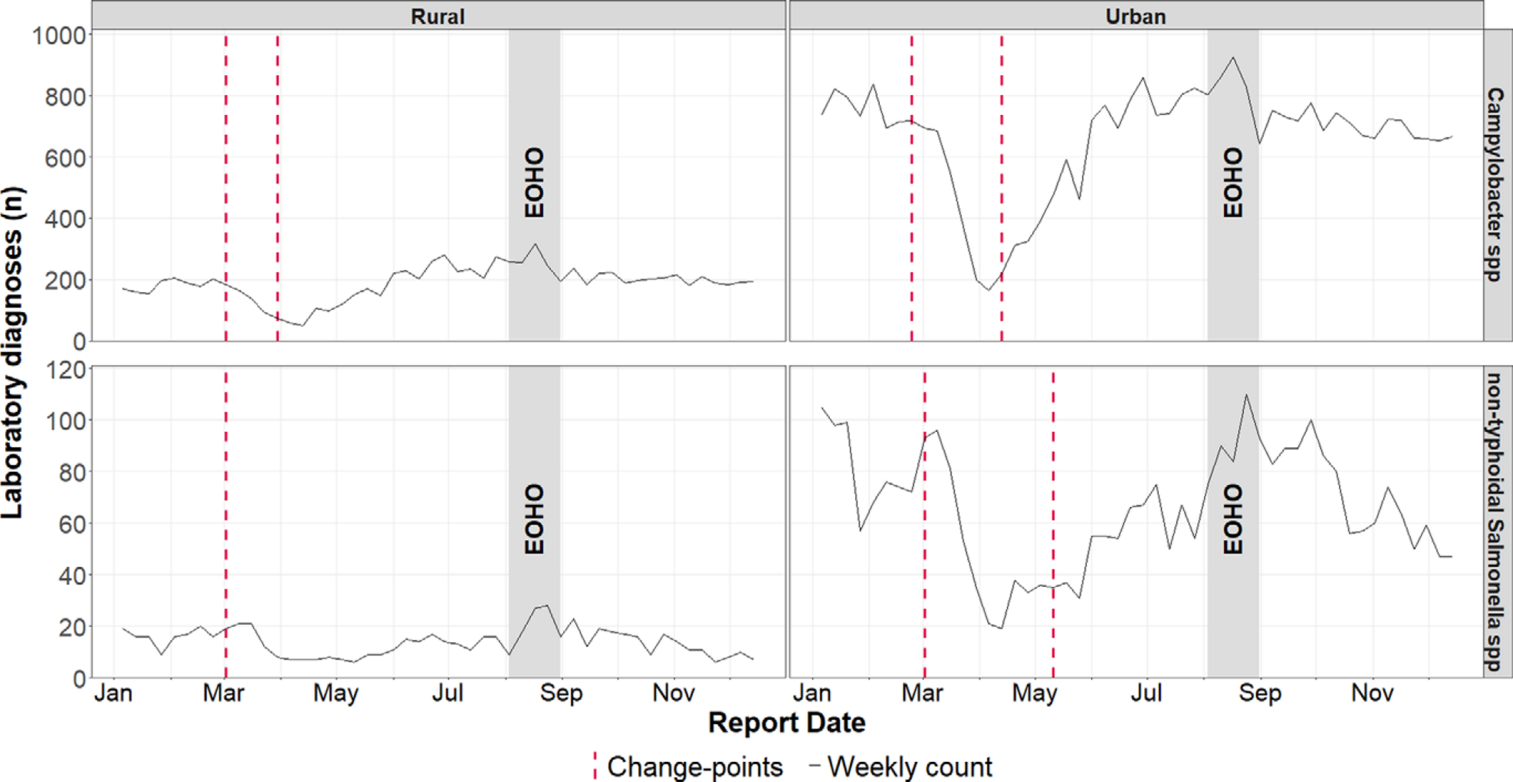
Weekly laboratory confirmed *Campylobacter* spp. and non-typhoidal *Salmonella* spp. infections in England during 2020, with identified change-points stratified by rural and urban residency. Change-points, identified using the Pruned Exact Linear Time method, reflect shifts in the mean and variance of residual deviations between observed rates and expected rates from a negative binomial generalized linear model, using population of each stratum as an offset variable.

Analysis of the dataset split by rural and urban residency, found no subsequent increase in cases of non-typhoidal *Salmonella* spp. among rural residents following the reduction in week 10 (02 March 2020) (Figure 5).

When stratified by IMD, change point in cases captured in 2020 for both *Campylobacter* spp. and non-typhoidal *Salmonella* spp. were inconsistent.

## Discussion

Our analysis did not identify a rise in the incidence of selected GI infections following the introduction of the EOHO scheme which encouraged dining out in August 2020. Instead, the observed reductive change points demonstrated the broader impact of NPIs implemented to mitigate COVID-19 on communicable diseases. While significant increases in GI infections were detected, these were aligned with the easing of NPIs rather than the EOHO scheme.

Public health measures aimed at controlling COVID-19 have had widespread unintended consequences, influencing the incidence of infectious diseases with varying transmission routes [35, 36]^.^ A key contributor to the decreases in GI observed in early 2020 will have been substantial changes in healthcare seeking behaviours as a result of the first national lockdown [37]. Notably, the impact of healthcare seeking behaviours on GI cases were less pronounced in subsequent lockdowns (5 November 2020 to 2 December 2020). These effects align with documented findings [16, 38] and are supported by our study. The observed increase in GI infections coinciding with the easing of the national lockdown [38], reinforces the plausibility that GI infection resurgence was influenced by changes in population movement [16, 38].

The temporal and spatial overlap in the implementation and easing of NPIs aimed at controlling COVID-19 posed a challenge in determining any specific impact of a single event on GI infections over this period in time. For instance, while restaurants were permitted to reopen on 4 July 2020 with social distancing, which was subsequently eased on 19 July 2020, the scheme did not begin until August 2020 [15]. While we did not identify any direct impact of the EOHO scheme, evaluating its effect with sufficient statistical power was challenging because of the difficulty in disentangling the scheme’s specific influence from other concurrent public health control measures.

Multifactorial COVID-19 control measures disrupted multiple communicable disease transmission routes beyond foodborne infections, including person-to-person spread and importation [35, 36]. The true impact of the EOHO scheme on GI infection transmission may have been confounded by these control measures, notably social distancing and improved hand hygiene [39]. Furthermore, it is plausible that genuine increases attributable to the scheme were offset by control measures that significantly disrupted transmission routes prior to its implementation. For example, substantial declines in international travel during 2020 likely reduced travel associated infections, inclusive of *Campylobacter* spp., *E. coli* O157, and non-typhoidal *Salmonella* spp. [2, 40]. In addition, high-risk settings associated with *E. coli* O157 outbreaks, specifically nurseries and petting farms [40], were closed as part of the national lockdown and did not reopen until June 2020 [15].

The seasonality of GI infections, typically peaking during the summer months [5], coincided with the introduction of the EOHO scheme. It is plausible that true increases in incidence of laboratory notifications following the scheme’s launch (between 10 August 2020 and 30 August) (Figure 1) may have been masked by natural seasonal trends.

National surveillance data does not capture all GI infections in the community as healthcare-seeking behaviours vary, which was notable during the COVID-19 pandemic [38, 41]. However, given the EOHO scheme lasted only four weeks, it is unlikely that significant changes in healthcare-seeking behaviours occurred.

While we identified change points for both *Campylobacter* spp. and non-typhoidal *Salmonella* spp., none were detected for *E. coli* O157. This may reflect limited statistical power, as annual case numbers for *E. coli* O157 were steadily declining while other GI infections remained stable prior to the COVID-19 pandemic. The observed decreasing trend in *E. coli* O157 cases over the study period aligns with broader epidemiological patterns [42]. Alternative modelling approaches may clarify whether changes in NPIs affected the incidence of *E. coli* O157.

The absence of a prompt resurgence in non-typhoidal *Salmonella* spp. cases among rural residents post-lockdown may be partly explained by differences in food consumption habits compared to urban areas. Rural populations generally consume less food prepared outside the home [43], which is notable given the majority of foodborne disease outbreaks can be attributed to Non-typhoidal *Salmonella* spp. [44]. Additionally, the rise of ‘dark kitchens’, (restaurants operating exclusively via delivery services) in urban areas, during COVID-19 [45] may have further influenced infection trends. These establishments have been associated with reduced trust in food safety and hygiene standards [46]. Managing food safety is a key challenge for such businesses, as they often operate in shared kitchens [47].

Our change point analysis allowed the data to determine where interruptions should be added, in contrast to prespecified interruptions. Compared to traditional methods which impose interruptions a priori, this approach objectively identified deviations between the model and the observed data. This offered an evidence-based assessment of the EOHO scheme’s impact on GI infections.

Our study used national data from a routine laboratory surveillance system, providing a large sample. The use of historical data over an extended period allowed for a robust baseline comparator to assess laboratory notifications in 2020 amid system changes. The inclusion of multiple GI infections offered a comprehensive evaluation of the EOHO scheme’s potential impact on the incidence of foodborne pathogens.

Stratifying the analysis by IMD reduced statistical power, limiting our ability to detect change-points. Moreover, IMD reflects socioeconomic conditions and is weighted on living environment, consequently making it confounded by urban and rural residency [27]. This confounding poses limits on our interpretation of the two characteristics independently.

Our study only analysed case data and did not review whether the EOHO scheme had an effect on the number of reported foodborne GI outbreaks. As not all GI infections are foodborne, incorporating such data may have provided a clearer assessment of the schemes impact. However, prior studies demonstrate that restaurant-associated foodborne outbreaks declined in conjunction with the introduction of NPIs [16].

Our models did not account for the phased implementation and easing of COVID-19 control measures beyond the first national lockdown, prior to the introduction of the scheme. These measures may have influenced GI infection trends (e.g., phased reopening of schools (1 June 2020), international travel corridors (8 June 2020), and reopening of restaurants and pubs (4 July 2020)).

## Conclusion

We found no evidence that the introduction of the EOHO scheme impacted the incidence of *Campylobacter* spp., *E. coli* O157, and non-typhoidal *Salmonella* spp. cases. The introduction of the first nationwide lockdown was followed by significant reductions in cases, with a prompt increase observed as enforceable restrictions eased. These findings emphasize the complex interactions between public health measures, behavioural changes, and the incidence of GI pathogens.

## Data Availability

The data used in this study is sensitive and was collected under Regulation 3 of the Health Service (Control of Patient Information) Regulations 2002, to process patient confidential information for national surveillance of communicable diseases. It is therefore not publicly available. Applications for relevant anonymized data should be submitted to the UKHSA Office for Data Release.
